# Preparation of Titania–Silica Composite Aerogel at Atmospheric Pressure and Its Catalytic Performance in the Synthesis of Poly (Butylene Succinate)

**DOI:** 10.3390/ma16093296

**Published:** 2023-04-22

**Authors:** Wenqi Zou, Hongli Bian, Jinjing Guo, Jun Xu, Baohua Guo

**Affiliations:** 1 Department of Chemical Engineering, Tsinghua University, Beijing 100084, China; 2 Beijing Center for Physical and Chemical Analysis, Institute of Analysis and Testing, Beijing Academy of Science and Technology, Beijing 100089, China

**Keywords:** Poly (butylene succinate), aerogel, catalysts, titanates, polycondensation

## Abstract

Titanates are widely used in the synthesis of polyesters, such as Poly (butylene succinate) (PBS), due to their excellent catalytic activity for polycondensation. However, the hydrolysis sensitivity of titanate and side reactions at high temperatures restrict the further improvement of the molecular weight of polyesters and lead to the high content of end carboxyl group content in the products. In this work, we prepared titania–silica composite aerogels with resistance to hydrolysis and large specific surface area, which were further explored as an efficient catalyst for polycondensation reactions. A series of titania–silica composite aerogel catalysts for PBS polycondensation were successfully prepared by the sol-gel method. The influence of a Ti/Si ratio on the surface morphology and structure of the aerogels was examined. Titania–silica composite aerogel exhibits the surface characteristics of high specific surface area and high Lewis acid content. The specific surface area of titania–silica composite aerogels can reach 524.59 m^2^/g, and the Lewis acid content on the surface can reach 370.29 μmol/g. Furthermore, the catalytic performance for the polycondensation reaction of PBS was investigated. The intrinsic viscosity of PBS synthesized by catalysis with the composite catalyst with a Ti/Si ratio of 9/1 reaches 1.74 dL/g, with the M_n_ of 7.72 × 10^4^ g/mol. The hydrolysis resistance stability of the titania–silica composite aerogel is greatly improved compared with traditional tetrabutyl titanate (TBT), and the end carboxyl group content of PBS is effectively reduced to lower than 30 mol/ton.

## 1. Introduction

Based on its excellent biodegradability, easy processing and comprehensive mechanical properties, PBS has been widely applied in the fields of packaging, agriculture and biomedicine [1,2,3]. At present, bio-based succinic acid (SA) and butanediol (BDO) has been commercialized and used as raw materials for PBS synthesis [4,5,6], which gives PBS a very broad market and application prospect [7]. However, due to the low melt strength, PBS usually needs high molecular weight to meet the processing and application requirements [8,9].

The preparation of high-molecular-weight PBS usually relies on the efficient removal of BDO by transesterification at high temperatures and high vacuum conditions in the polycondensation stage. On the one hand, with the decrease of the end hydroxyl content in the reaction system, the chain growth reaction and pyrolysis reaction rate finally reach a balance, and the molecular weight will be difficult to increase further [10]. On the other hand, this reaction environment also leads to some side reactions, such as the pyrolysis of polyester, resulting in the rise of the end carboxyl group content and the yellowing of the product [11], which will ultimately have a negative impact on the performance of PBS. To solve the problems, proper selection of catalytic systems can effectively improve catalytic efficiency and reduce side reactions. Therefore, further development of new efficient and environmentally friendly catalyst systems has become a key issue in the preparation of polyesters such as PBS.

In fact, compounds of various main groups and transition metal elements can catalyze the synthesis of polyesters, such as tin (Sn), antimony (Sb), germanium (Ge), titanium (Ti), aluminum (Al) and rare earth element compounds with empty orbitals, etc. [11,12]. Considering the cost and performance, Ti-based catalysts with high catalytic activity, no heavy metals and moderate cost are the research hotspots in the catalytic synthesis of polyesters from diacids and diols [13]. Titanate compounds and their complexes are commonly used in Ti-based polyester polycondensation catalysts, but they generally have problems, such as hydrolysis sensitivity, fast side reaction rate and yellowing of the prepared polyester products [14]. Titanium dioxide (TiO_2_) and titanium oxide composites have better stability and fewer side reactions than titanates [15]. At present, the research on titanium composite oxide catalysts mostly focuses on the molecular sieve structure and the large specific surface area. The ordered pore structure of molecular sieves was used to improve the catalytic efficiency. Hwang et al. [16] synthesized PBS composites with titanium–silicon composite (TS-1) zeolite as the catalyst and studied the effect of TS-1 zeolite on the physical properties of PBS. TS-1 as a catalyst and filler can improve the tensile strength and elongation at the break of PBS. Thakur et al. [17] prepared a series of titania–silica composite oxide catalysts modified by different organic groups through co-precipitation of tertbutyl titanate and siloxanes with different organic groups, and PET was synthesized via the prepared catalysts. The experimental results showed that the titanium–silicon composite catalyst modified with specific organic groups could play an excellent catalytic role in the polycondensation reaction while reducing the amount of titanium. However, the molecular sieve structure usually needs to be sintered at a high temperature to remove the templating agent. A more easily prepared and efficient titanium–silicon composite catalytic system is urgently needed for further development.

Aerogel is a kind of solid-phase material with a stable internal network structure and multi-level pore structure formed by stacking nano-scale particles [18]. By adjusting the preparation process and element composition, the physical and chemical properties of the aerogel surface can be controlled to achieve different functions [19,20]. The catalytic effect of titania–silica composite aerogels on the polycondensation of polydiacid dialcohol esters has not been extensively studied. The high specific surface area of titania–silica composite aerogels, coupled with the enhanced Lewis acidity of Ti atoms through Si-doping [21,22], makes them ideal candidates for a highly active and stable Ti-based polyester catalyst system. However, there are limited reports on the catalytic effect of titania–silica composite aerogels on the polycondensation of diacids and diols. Furthermore, the chemical environment and dispersion state of Ti and Si atoms on the surface of their composite oxides can affect the number and strength of Lewis acid sites on the material surface, which in turn affects the catalytic activity of the ester-exchange reaction.

Herein, in order to overcome the problems of hydrolysis sensitivity and side reactions of traditional titanate catalysts, a series of titania–silica composite aerogels with different chemical compositions were prepared by the atmospheric pressure drying method. The morphology and structure of the aerogel were controlled by adjusting the ratio of Ti to Si. The prepared aerogel was used as a transesterification catalyst to catalyze the polycondensation of PBS, and the polycondensation kinetics was determined as well. The structural characteristics of the titania–silica composite aerogels and its catalytic activity for the polycondensation reaction of PBS were further explored, which provided a new idea for the structural design of hydrolysis-resistant and efficient catalysts for polyester synthesis.

## 2. Materials and Methods

### 2.1. Materials

TBT and ethyl orthosilicate (TEOS) were supplied by Shanghai Aladdin Biochemical Technology Co., Ltd., Shanghai, China. Absolute ethanol (EtOH) and N,N-dimethylformamide (DMF) were purchased from Shanghai McLean Biochemical Technology Co., Ltd, China. Acetic acid (HAc) and hydrochloric acid (HCl) were purchased from Beijing Chemical Factory. Succinic acid (SA) and butanediol (BD) were obtained from Beijing J&K Scientific Technology Co., Ltd., Beijing, China. The purity of the above reagents is analytical reagent.

### 2.2. Preparation of Titania–Silica Composite Aerogels

**Figure 1:**
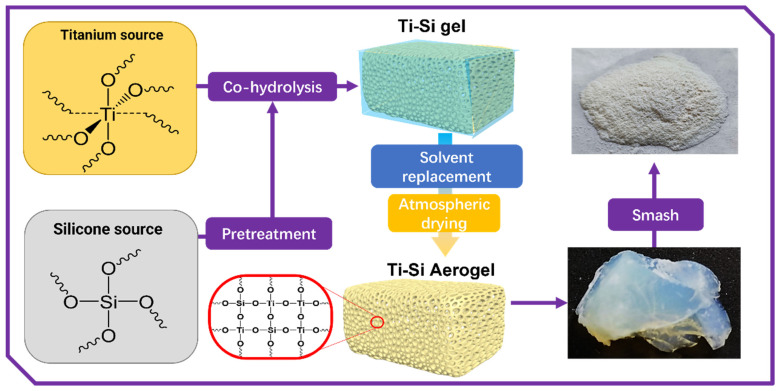
The schematic diagram of the preparation process of the titania–silica composite aerogel.

The following will briefly describe the preparation process of the titanium–silicon composite aerogel by taking the preparation process of the aerogel sample with a titania–silica molar ratio of 9:1 as an example. The schematic diagram of the process is shown in Figure 1.

#### 2.2.1. Pre-Hydrolysis of Tetraethyl Silicate

First, 2.64 mL of EtOH was placed in a 50 mL beaker, and 1.72 g of TEOS was dispersed in it and stirred for 30 min. Then, 0.59 mL deionized water and 0.14 mL HCl solution with a 3.0 pH value was added dropwise into the solution through a dropping funnel. The above-mixed system (solution A) was magnetically stirred at room temperature for 24 h before use.

#### 2.2.2. Preparation of Titania–Silica Composite Alcohol Gels

First, 69 g of EtOH and 9 g of HAc were placed in a 250 mL beaker for mixing, and then 27 g of TBT was added and stirred for 30 min to obtain solution B. Solution A was slowly dropped into solution B through the dropping funnel and kept stirring for 30 min. Then 6 mL deionized water and 1 mL DMF was added dropwise to the obtained mixed solution. After stirring for 30 min, the system was placed in a water bath at 35 °C. After the system was gelatinized, it was continued to age at 35 °C for 24 h to obtain a titania–silica composite alcohol gel for use.

#### 2.2.3. Preparation of Titania–Silica Composite Aerogels

The alcohol gel prepared in Section 2.2.2 was immersed in the solution with the mass ratio of deionized water/ethanol of 1/1 at room temperature for aging for 12 h, then the same gel was quickly taken out from the system and soaked in the isopropanol/n-hexane mixed solution with the volume ratio of 4/1, 2/1, 1/1, 1/2 and 1/4 for 12 h at 55 °C. Finally, the gel was soaked in n-hexane for 24 h in order to minimize the potential damage to the gel network structure caused by the rapid solvent replacement. By employing solvent exchange, the highly polar H_2_O and alcohol were replaced with low-polarity n-hexane, reducing the damage to the aerogel’s pore structure caused by capillary action during the subsequent drying process. After the solvent exchange was completed, the gel was placed in a convection oven and dried at 60, 80 and 100 °C under atmospheric pressure for 4, 2 and 2 h, respectively. As the solvent molecules gradually evaporated from the gel’s pores, gas molecules replaced them within the gel network, ultimately resulting in a titania–silica composite aerogel.

Titania–silica aerogels with different molar ratios of Ti/Si and titanium dioxide aerogel (TiO_2_-AG) were also processed in the same way. Titania–silica composite aerogel with a Ti/Si molar ratio of 9/1 was named TS91, and samples with Ti/Si molar ratios of 8/2, 7/3, 6/4 and 5/5 were designated as TS82, TS73, TS64 and TS55, respectively.

### 2.3. Preparation of PBS

First, 24.75 g BDO and 29.5 g SA were added to a 250 mL three-necked flask and connected to the nitrogen interface, mechanical stirring and water separator. After that, the system was successively esterified at 140 °C for 2 h, 150 °C for 1 h, 160 °C for 1 h, 170 °C for 1 h and 190 °C for 0.5 h under a nitrogen atmosphere. After the collected water in the water separator reached more than 90% of the theoretical value and remained unchanged, the system was cooled to 150 °C, and an 85 μL catalyst (titania–silica composite aerogel, TiO_2_-AG, TBT) was added into the system and stabilized for 15 min. Then, the system was evacuated to below 100 Pa and rapidly heated to 230 °C for polycondensation. After the system was reacted at 230 °C for 2 h, the reaction was completed, and the prepared PBS was collected. PBS synthesized by different catalysts was denoted as PBS-x, where x represents catalysts.

### 2.4. Characterization and Measurement

X-ray photoelectron spectroscopy (XPS): The surface elemental composition of the samples was characterized by k-alpha type (Thermo Fisher Scientific, Waltham, MA, USA). X-ray photoelectron spectroscopy and monochromatic Al Ka radiation were used.

Specific surface area analysis: The specific surface area of the samples was obtained by a Brunauer Emmett Teller (BET) analysis based on the nitrogen adsorption-desorption isotherm measured at 77 K. The test equipment was an asap-2460 physical adsorption analyzer (Micromeritics Instrument Corporation, Norcross, GA, USA).

Micro morphology: the surface structure of the sample was characterized by scanning electron microscopy (SEM, Japan Electron Optics Laboratory Co., Ltd., Tokyo, Japan, jsm-7800f).

Pyridine infrared (Py-IR): The content and type of acid on the surface of the samples were characterized by the sensor 27 Fourier infrared spectrometer of Luke spectroscopic instruments. The pretreatment temperature of the samples was 100 °C under vacuum for 2 h to remove the adsorbed impurities on the surface of the samples. After that, the temperature of the adsorption cell was raised to 200 °C and kept for 30 min for sampling and scanning.

Ammonia temperature programmed desorption (NH_3_-TPD): Auto Chem 2910 temperature programmed chemisorption instrument was used to characterize the acid properties of the catalyst. The pretreatment temperature of the sample was 100 °C under vacuum for 2 h to remove the adsorbed impurities on the surface of the sample. Then, NH_3_ was introduced into the quartz reaction tube at 100 °C for 30 min adsorption. Finally, the signal of the TCD detector was recorded in the temperature range of 50~600 °C and processed with corresponding software. The heating rate was 10 °C/min.

Intrinsic viscosity: Using the 1,1′,2,2′-tetrachloroethane/phenol-mixed solution with a mass ratio of 1/1 as a solvent, a PBS solution with a concentration of 0.005 g/mL was prepared. The “one-point method” was used to measure the efflux time, and the intrinsic viscosity of the corresponding PBS sample was calculated by the following formula (1):

(1) $$\left[\eta \right]=\frac{{\left\{2\left[\frac{t}{t_{0}}-ln\left(\frac{t}{t_{0}}\right)-1\right]\right\}}^{\frac{1}{2}}}{C}\;$$

where [*η*] is the intrinsic viscosity of the sample in dL/g; *t*_0_ is the outflow time of pure solvent in s; *t* is the outflow time of PBS solution in s; and *c* is the concentration of PBS solution in g/dL. The number-average molecular weight M_n_ of the PBS samples was calculated by the Berkowitz Equation (2) [22]:

*M_n_* = 3.29 × 10^4^ [*η*]^1.54^ (2)

## 3. Results and Discussion

In order to provide a theoretical basis for exploring the performance differences between different titania–silica composite aerogel catalysts, the structural characterization of the aerogel samples with different Ti/Si ratios was first carried out.

### 3.1. Surface Morphology and Structure of Titania–Silica Composite Aerogels

**Figure 2:**
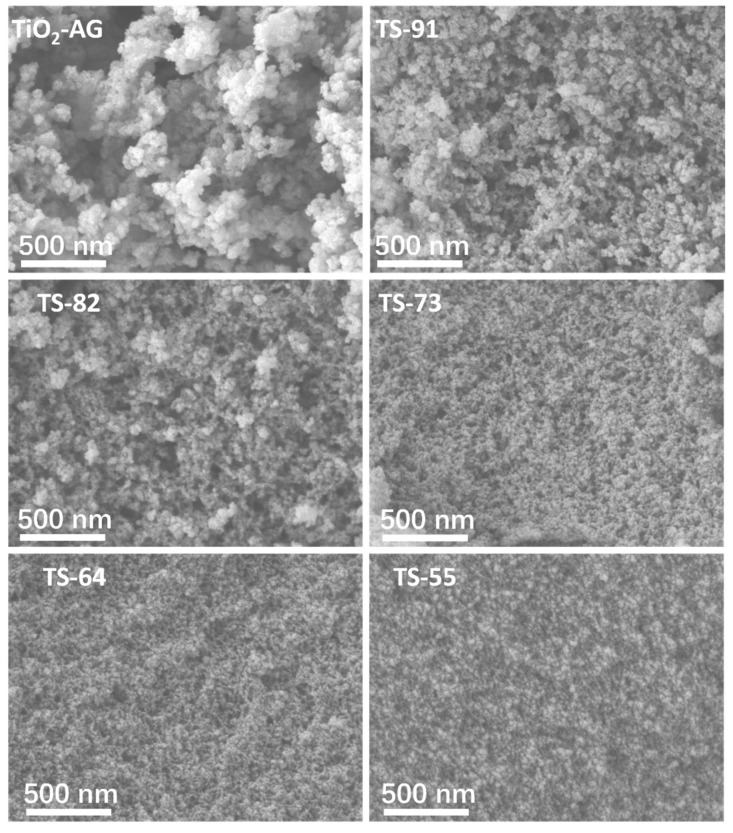
Surface morphology of aerogel samples with different Ti/Si ratios.

The surface morphology of the aerogel samples with different Ti/Si ratios was analyzed by SEM, and the results are shown in Figure 2.

TiO_2_-AG shows a coral structure formed by the accumulation of irregular particles, in which a relatively significant agglomeration morphology is formed between the particles. Macropores are formed between the particle clusters, while the irregular surface of particles and the gaps between particles lead to potential micropores and mesoporous morphology. During the gelation process, Ti/Si sources are first hydrolyzed and cross-linked to form primary microgel particles and then turn into a sol system. The primary gel particles gradually grow and collide with each other to form clusters [23]. Finally, the clusters are cross-linked with each other to form the final three-dimensional gel network structure composed of microgel clusters [24]. After solvent replacement and drying at atmospheric pressure, the above three-dimensional network structure is still largely retained, thereby forming aerogels [25].

With the introduction of the Si element into the titania–silica composite aerogel, the diameter of the primary particles constituting the aerogels is significantly reduced. The agglomeration between particles is significantly weakened, and the surface pore size distribution is more uniform than that of the TiO_2_-AG. With the increase of the Si content, the pore structure on the surface of the materials further tends to be uniform and dense. When the Si content exceeds 30% of the total content of Ti and Si, the macroporous structure on the surface of the material is significantly reduced.

**Figure 3:**
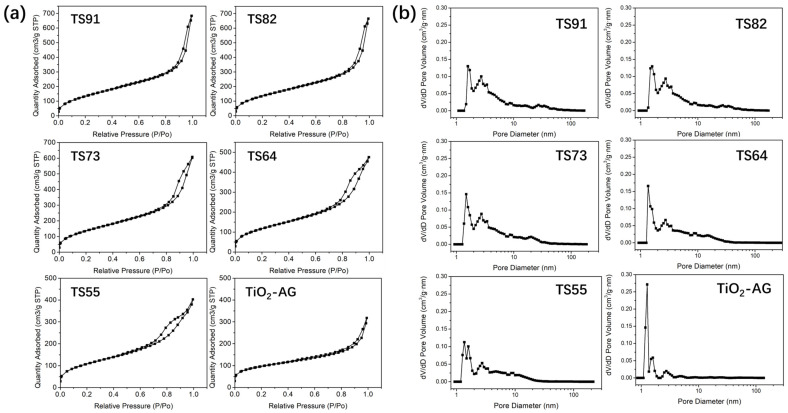
Nitrogen adsorption/desorption isotherms (**a**) and pore size distribution (**b**) of composite aerogel samples with different Ti/Si ratios.

In order to further describe the surface morphology of the prepared aerogels, nitrogen adsorption/desorption analysis was performed on the different titania–silica composite aerogels. Figure 3a shows the nitrogen adsorption/desorption isotherms of the samples, and Figure 3b shows the pore size distribution of the corresponding samples, with the relevant specific surface area data listed in Table 1. It can be seen that all the samples exhibit type-IV isotherms [26]. There is an inflection point in the relative pressure range of 0.05–0.1 P/P0 for the aerogels. Before the inflection point, the nitrogen adsorption content rises rapidly with the increasing relative pressure, which corresponds to the single-layer adsorption of nitrogen in the microporous structure on the surface of the material. Saturated monolayer adsorption is reached at the inflection point. With the further increase of the relative pressure, the nitrogen adsorption capacity of different samples shows an approximately linear rising range in the range of 0.1–0.8 P/P0, which is related to the capillary condensation phenomenon of nitrogen in the mesopores and the multi-layer adsorption in the micropores [27]. In the high relative pressure range, the rising of the isotherm corresponds to the existence of macropores. The hysteresis loop indicates the existence of slit-shaped pores and the capillary condensation phenomenon therein [28,29].

TiO_2_-AG generally shows lower nitrogen adsorption and a smaller specific surface area, which may be due to the rapid hydrolysis rate of TBT alone in the early stage of gel formation, resulting in the agglomeration of primary TiO_2_ microgel particles [30]. Compared with other samples, the isotherms of TS-91, TS-82 and TS-73 show larger slopes in the medium pressure range, indicating that these samples have relatively more mesoporous structures. The larger rise of the isotherm in the high-pressure region indicates a larger pore size than that of other samples. The SEM morphologies (Figure 2) demonstrate that the introduction of Si significantly limits the particle size of the primary gel particles in the system. The dehydration condensation between silanols and the formation of cross-linked networks are slower than the hydrolysis and condensation between TBT molecules. Therefore, the access/adsorption of Si gel network segments slows down the growth rate of the primary gel particles formed in the system [22,31,32]. As a result, large quantities of primary gel particles of titania–silica composite aerogels lead to smaller particle sizes than that of TiO_2_-AG, resulting in a large specific surface area and many cracks between particles. For TS-64 and TS-55, the nitrogen adsorption capacity of the samples in the medium and high-pressure range decreases with the increase of Si content, and the slope of the adsorption curve also reduces, which corresponds to the decrease of mesopores and macropores of the samples. In addition, when the Ti/Si in the sample reaches 8/2 with the further increase of the Si ratio, the pore size and pore volume of the sample both show a downward trend. The slit-shaped pores are dominant in titania–silica composite aerogels. The size of primary gel particles is reduced with the increased Si content, which may be attributed to relatively densely formed smaller slit pores, leading to the smaller pore size and pore volume.

**Figure 4:**
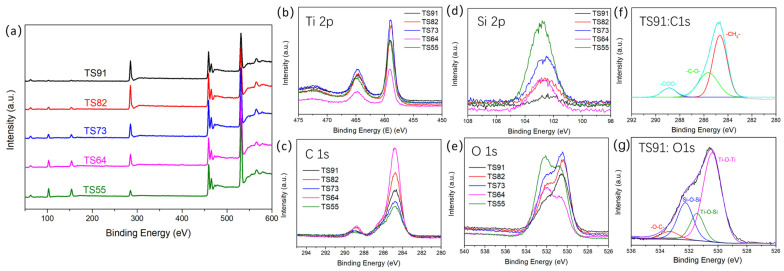
XPS full spectrum (**a**) and high-resolution spectra (**b**–**e**) of composite aerogels with different Ti/Si ratios. (**f**,**g**) are the high-resolution XPS spectra of C 1s and O 1s of sample TS91, respectively.

XPS was utilized to elucidate the chemical composition and structure of titania–silica composite aerogel, which has a significant impact on the catalytic performance of the materials, as shown in Figure 4 and Table 2. Ti, O, Si and C elements are detected in all samples (Figure 4a), and the ratio of Ti and Si elements in the raw materials during the feeding step effectively affects the surface elemental composition of the prepared titania–silica composite aerogels (Table 2). For all the samples, the atomic ratio of Ti/Si on the surface of the aerogels is slightly lower than the theoretical atomic ratio of Ti/Si in raw materials, indicating that Si atoms tend to be enriched on the surface of the microgel. The probable reason is that during the formation process of the primary microgel, the Ti source with a faster hydrolysis/gelation rate first forms the core part of the microgel, and then the Si gel network fragments begin to access and adsorb on the Ti gel surface.

Titania–silica composite aerogels show the signal peak of Ti 2p near 460.0 eV and 465.0 eV (Figure 4b), which represents the typical 2p3/2 orbital characteristic peak and 2p1/2 orbital peak of IV-valent Ti [22,33,34], respectively. Namely, there is only one chemical structure of Ti in titania–silica composite aerogels, corresponding to the -Ti-O- bond. The high-resolution spectra of Si 2p for all the samples exhibit a single peak around 102.5 eV (Figure 4d), indicating that Si atoms mainly exist in the form of polysiloxane in titania–silica composite aerogels.

As shown in Figure 4e, the peak splitting intensity of O 1s around 532.6 eV is significantly enhanced with increasing Si content in the aerogels. In the case of TS91, the O 1s peak can be divided into three peaks, corresponding to the structure 530.5 eV (-Ti-O-Ti-), 533.3 eV (-C-O-) and 532.3 eV (-Si-O-Si-) [33,35] and 531.5 eV (-Ti-O-Si-) [36]. Similarly, the characteristic peaks of C 1s have similar peak shapes. Taking TS91 as an example, the fitted C1s spectrum of TS91 exhibits three peaks at 284.7 eV (-C-C-), 286.2 eV (-C-O-) and 289.1 eV (-O-C=O), respectively [33,37], corresponding to the alkoxy structure and trace amount of acetic acid residue added during the preparation process of aerogels. It can be seen that there are plentiful alkoxy structures on the surface of the aerogels.

For the transesterification of polyester in the polycondensation stage, the strength and amount of Lewis acid on the catalyst surface have a direct impact on its catalytic activity. For the characterization of catalyst acidity, Py-IR technology and NH_3_-DPT technology are usually combined. Among them, Py-IR characterization focuses on different acid types and the corresponding intensities. With the pyridine molecule as the probe molecule, the different infrared characteristic peak positions of the pyridine molecule combined with Brönsted acid (B acid) and Lewis acid (L acid) on the catalyst can be used to obtain the information on B acid, L acid and total acid content on the catalyst, respectively [38]. NH_3_-DPT uses an ammonia molecule as a probe molecule to combine with the acid site in the catalytic material and distinguishes between strong and weak acids according to the desorption temperatures of the ammonia molecule.

**Figure 5:**
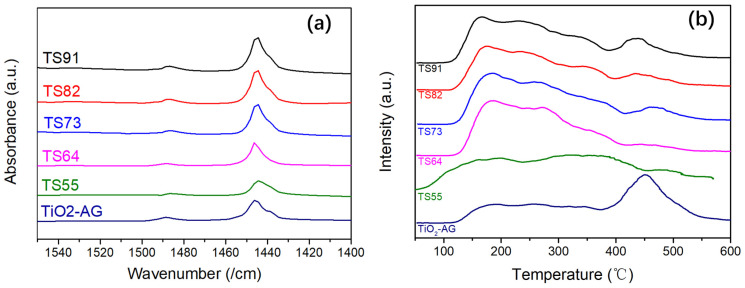
Py-IR (**a**) and NH_3_-DPT (**b**) analysis of composite aerogels with different Ti/Si ratios.

In order to investigate the acid species and their distribution on the surface of the aerogels, Py-IR characterization was employed to analyze aerogel catalysts with various Ti/Si ratios. Figure 5a displays the Py-IR characterization results of the titania–silica aerogels, and the corresponding data are presented in Table 3. According to previous studies, the infrared spectral peaks near 1450 cm^−1^ and 1540 cm^−1^ in the spectra are associated with the L acidic centers and B acidic centers of the catalyst, respectively. The infrared spectrum peak near 1490 cm^−1^ is attributed to the synergistic effect of L acidic centers and B acidic centers [39]. Figure 5a exhibits strong infrared peaks around 1450 cm^−1^, corresponding to the L acidic centers. B acidic centers are present in trace amounts, as evidenced by the minor peaks around 1540 cm^−1^. Figure 5b depicts the NH_3_-DPT spectra of titania–silica composite aerogels and TiO2-AG. The desorption signal peaks of NH_3_ molecules between 100 and 600 °C correspond to the gradual desorption behavior of NH_3_ molecules adsorbed on the acidic sites of the catalyst surface as the temperature increases. Furthermore, the signal peaks within the ranges of 100–300 °C, 300–450 °C and 450–600 °C are attributed to the adsorption of NH_3_ on weak acidic centers, moderately strong acidic centers and strong acidic centers of the sample, respectively [39,40]. It is noteworthy that the number of strong acid sites on the material surface significantly decreases with increasing silicon content, suggesting that the interaction force between titanium atoms and NH_3_ molecules on the surface is weakened after coordination. This phenomenon might be attributed to the introduction of silicon and the potential Ti-O-Si bond, where the charge imbalance and steric hindrance effect generated by the silicon atom reduce the L-acid strength of titanium atoms.

As indicated in Table 3, L acidic centers are the predominant surface acidic centers of all aerogel samples, which originates from the electron pair accepting capability provided by the empty orbital of Ti atoms on the surface. The presence of a small content of B acidic centers may result from the trace acetic acid residue during the aerogel preparation process. The surface acidic center content of TiO_2_-AG is relatively low; for instance, the L acidic center content of TiO_2_-AG is only 221.29 μmol/g. However, the surface acidic centers’ density of TiO_2_-AG is not lower than that of titania–silica composite aerogels, confirming that the surface acidic centers’ content of TiO_2_-AG is primarily limited by its low specific surface area (332.86 m^2^/g). In comparison to TiO_2_-AG, the L acidic center content on TS91’s surface increases significantly to 370.29 μmol/g. As the Si content in the system increases, both the content and density of L acidic centers on the surface of the samples decline rapidly. In conjunction with BET characterization results (Table 1), the incorporation of a small amount of Si substantially enhances the specific surface area of the aerogels, which facilitates the exposure of L acidic centers on the material’s surface, thereby considerably improving the L acidic centers’ content. With a further increase in Si content, the specific surface area of the material diminishes rapidly. Simultaneously, the increase in surface siloxane structure dilutes the L acidic centers, leading to a decrease in the density and content of L acidic centers on the surface of the materials. It is important to mention that with increasing Si content, the amount of strong acidic centers on the material’s surface notably declines (Figure 5b), indicating that the interaction force between surface Ti atoms and NH_3_ molecules is weakened. This observation may be ascribed to the introduction of Si, which leads to the potential charge imbalance of the -Ti-O-Si- bond and the steric hindrance effect produced by Si reduces the L acidic center strength of the associated Ti atoms.

### 3.2. Catalytic Activity of Titania–Silica Composite Aerogels for the Polycondensation of PBS

PBS was synthesized via a two-stage method (esterification and polycondensation) using titania–silica composite aerogel as the catalyst. The catalytic activity of the catalyst can be reflected by the intrinsic viscosity and molecular weight of the prepared PBS, as shown in Table 4.

PBS synthesized without catalyst exhibits an intrinsic viscosity of only 0.63 dL/g, while the intrinsic viscosity of PBS synthesized by TiO_2_-AG catalyzed is 1.14 dL/g, proving the valid catalyst effect of Ti-based aerogel for the synthesis of PBS. With the introduction of Si in the aerogel catalyst, the intrinsic viscosity of the synthesized PBS is greatly improved. The intrinsic viscosity of the PBS synthesized by TS91 can reach 1.74 dL/g, indicating the high catalytic activity of TS91. With the continuously increasing Si content in the catalyst, the intrinsic viscosity of the prepared PBS gradually decreases, suggesting the decreasing catalyst activity of titania–silica composite aerogel with rising Si content.

When the organometallic compound catalyzes the synthesis of polyester, the catalytic process includes the following steps [10]. We take titanate catalyst as an example. First, the hydroxyl group at the end of the molecular chain in the reaction system will undergo a ligand exchange reaction with the organic group directly connected to the titanium atom (usually alkoxy or carboxylic acid group, i.e., ligand). Second, the active titanium center will be connected to the chain end of the molecular chain to form an active species. Third, the Ti atom in the center of the active species coordinates with the carbonyl O atom in another molecular chain through its empty orbit. The coordination makes the carbon–oxygen bond electron cloud in the carbonyl group shift to the O atom side, and the corresponding carbon atom is more likely to be attacked by the nucleophilic agent. Fourth, the titanium alcohol hydroxyl group performs a nucleophilic attack on the carbonyl carbon to form a new polymer chain, while the coordination of the Ti atom with carbonyl oxygen is released to form a new active species so that the reaction can be repeated. To sum up, in this reaction system, the catalyst is actually catalyzing the transesterification reaction between molecular chains, so the rate of ligand exchange and coordination/dissociation between the Ti core and carbonyl O atom will affect the activity of the catalyst [10]. In the polycondensation process, when this transesterification reaction occurs at the end of the molecular chain, the small end group molecules produced by the exchange will be separated from the reaction system under high temperature and vacuum conditions so as to realize the positive growth of polyester molecular weight.

For the prepared titania–silica composite aerogels, a large number of alkoxy structures connected with Ti atoms on the surface provide the possibility of ligand exchange with the hydroxyl group at the end of the molecular chain. Meanwhile, a large number of L acidic centers on the surface provide space for ligand exchange. By comparing the information in Table 3 and Table 4, it can be seen that there is a strong correlation between the characteristic viscosity of the synthesized PBS and the Lewis acidic centers’ content on the catalyst surface, with a higher Lewis acidic centers’ content corresponding to a higher characteristic viscosity of PBS. For TiO_2_-AG, the low content of L acid on the surface limits the number of active sites that can coordinate with carbonyl O atoms, so the catalytic activity is relatively low. When Si is introduced to titania–silica composite aerogels, with the increasing specific surface area, more L-acid active sites are exposed and greatly improve the catalytic activity. However, with the further increase of Si element content, the L-acidic centers and alkoxy groups on the surface of the catalyst decrease, resulting in the rapid decline of catalytic activity. It is worth mentioning that the surface L acidic centers’ content of TS64 and TiO_2_-AG are similar, 208.83 μmol/g and 221.29 μmol/g, respectively. However, TS64 shows higher catalytic activity than TiO_2_-AG. The intrinsic viscosities of the PBS synthesized by TS64 and TiO_2_-AG are 1.35 and 1.14 dL/g, respectively. The L acidic centers on the surface of TiO_2_-AG mainly exist as strong acidic centers, while the surface acidity of TS64 is obviously weak (Figure 5b). This phenomenon indicates that the strong L acidic centers in the system may have a negative impact on the catalytic activity. There is a strong lone pair electron interaction appears between the strong L acidic centers and the carbonyl O atom during coordination, which may cause difficulty in releasing the coordination after the transesterification reaction, which hinders the site from participating in the subsequent catalytic reaction and results in the reduction of catalytic activity.

In summary, the prepared titania–silica composite aerogels can effectively catalyze the polycondensation of PBS. Among them, TS91, with a Ti/Si molar ratio of 9:1, presents the highest catalytic activity.

### 3.3. Reaction Kinetics of PBS Polycondensation Catalyzed by Titania–Silica Aerogel

In order to deeply understand the catalytic activity of titania–silica composite aerogel for the polycondensation reaction of PBS, five temperatures, 220, 225, 230, 235 and 240 °C were selected to investigate the kinetics of the polycondensation reaction catalyzed by TS91. The traditional titanate catalyst TBT was used as the benchmark.

Since the vacuum degree is less than 100 Pa, the study of polycondensation kinetics does not consider the occurrence of side reactions. In addition, it is assumed that all the chain ends of PBS are hydroxyl groups. The polycondensation of PBS satisfies the calculation of the second-order reaction Equation (3) proposed by Rafle [22,41].

(3) $$-\frac{dC_{OH}}{dt}=KC_{OH}^{2}$$

After integration, the following Equation (4) can be obtained:

(4) $$\frac{1}{C_{t}}-\frac{1}{C_{0}}=Kt$$

Among them, *C_OH_* is the terminal hydroxyl group concentration of PBS, in units of mol/L, *C_t_* is the terminal carboxyl group concentration at time t after the start of vacuum polycondensation, and *C_0_* is the terminal hydroxyl group concentration at the beginning of the reaction. At the same temperature, samples were taken out every 25 min for an intrinsic viscosity characterization. The viscosity average molecular weight *M_v_* is calculated according to Formula (5):

(5) $$\left[\eta \right]=kM_{v}{}^{\alpha }$$

where [*η*] is the intrinsic viscosity and *M_v_* is the viscosity-average molecular weight of the sample. The Mark–Houwink constant *k* and *α* are 1.71 × 10^−4^ and 0.79, respectively. Afterward, the hydroxyl concentration of the reaction system at time t can be calculated via formula (6):

(6) $$C_{t}=\frac{2}{M_{v}}$$

Linear fitting of *M_v_* and *t*:

(7) $$M_{v}=Kt+M_{0}$$

**Figure 6:**
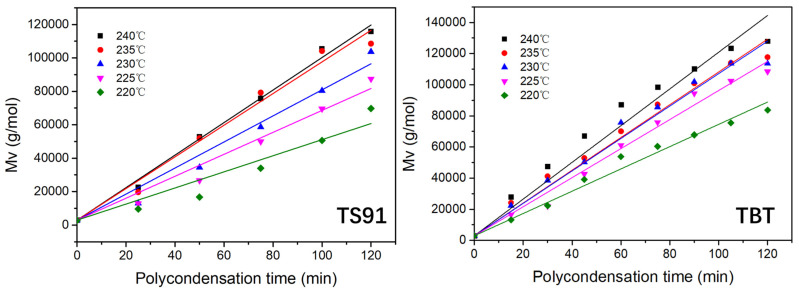
Linear fitting of Mv-polycondensation time of PBS catalyzed by TS91 and TBT at different temperatures.

For different catalytic systems, *M_v_* and *t* measured at various polycondensation temperatures were fitted (Figure 6), and the fitting equations are summarized in Table 5.

**Figure 7:**
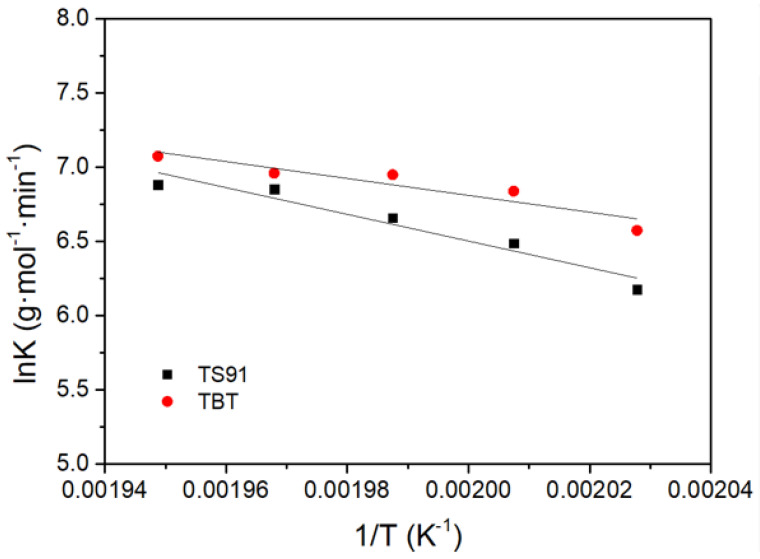
Linear fitting of lnK versus 1/T in the polycondensation reaction of PBS catalyzed by TS91 and TBT.

The slopes of the fitting curves give the reaction rate constants *K* at the studied reaction temperatures T. According to the Arrhenius Equation (8) (Figure 7),

(8) $$lnK=lnA-\frac{E_{a}}{RT}$$

where *A* is the prefactor, E_a_ is the activation energy of the reaction and *R* is the molar gas constant, 8.314 J/(mol·K).

The activation energy (Ea) of TS91 and TBT, which are 74.76 kJ/mol and 47.28 kJ/mol, respectively, plays a significant role in determining the catalytic activity of these compounds. TS91 exhibits good catalytic activity for polycondensation reactions.

One possible explanation for the higher activation energy of TS91 is the increased steric resistance resulting from the introduction of silicon. The silicon atom present in TS91 might lead to a more crowded and hindered environment around the catalytic center. This steric hindrance can impede the approach of reactants and slow down the reaction, thus leading to a higher activation energy requirement.

In addition to the steric effects, the dispersion of the catalyst in the reaction system also plays a crucial role in determining the catalytic activity. TBT can be dispersed in the reaction system in a homogeneous form during the polycondensation reaction of PBS. In this case, all the titanium catalytic centers can theoretically participate in the reaction, leading to a more efficient catalytic process. On the other hand, TS91 in the form of an aerogel can only be dispersed in a heterogeneous form. Consequently, only surface atoms can play a catalytic role, limiting the overall efficiency of the catalyst. This restricted participation of the catalytic centers in TS91 contributes to its lower catalytic activity as compared to TBT. To further enhance the catalytic activity of TS91, it is possible to try further optimizing its preparation process, such as the preparation of a titanium-silicon homogeneous catalytic system and promoting the formation of titanium–silicon microgels by the water generated during esterification.

Although TS91 exhibits a slightly lower catalytic activity than TBT, it shows better hydrolysis resistance and thermal stability than TBT. As shown in Table 4, when the catalyst was added after the esterification reaction, the intrinsic viscosity of PBS synthesized by TBT was 1.89 dL/g, which is higher than 1.74 dL/g by TS91. However, when the catalyst is added together with the other raw materials before the esterification reaction, the intrinsic viscosity of PBS synthesized by TBT can only reach 1.26 dL/g while TS91 can reach 1.78 dL/g under the same reaction temperature and time. During the esterification process, a large number of generated water molecules promote the hydrolysis of TBT, thus forming solid-phase TiO_2_ particles, so the catalytic activity significantly decreases. Compared with TBT, TS91 shows good hydrolysis stability because it is the product of hydrolysis. At the same time, the content of terminal carboxyl groups in PBS catalyzed by TBT reaches 51.68 mol/t. Under the same conditions, the content of terminal carboxyl groups in PBS prepared by TS91 is only 25.45 mol/t, indicating that TS91 can inhibit side reactions, such as thermal oxidative degradation and thermal decomposition during polycondensation. In addition, combined with the changes in the b value in the CIELAB color space given in Table 4 (an increased b value indicates that the material is comparatively more yellow), the b value of PBS synthesized by TBT reached 4.61, while the b value of PBS prepared by TS91 dropped to 1.97. The use of an aerogel catalyst also effectively alleviated the yellowing of PBS products (usually caused by thermal oxidative degradation during polycondensation), which also reflected the inhibition of this series of catalysts on related side reactions.

## 4. Conclusions

In order to overcome the problems of hydrolysis deactivation and side reactions of traditional titanate catalysts, a series of titania–silica composite aerogels with different Ti/Si ratios were prepared at atmospheric pressure. The effects of the Ti/Si ratio on the surface morphology, specific surface area, chemical composition and the type and content of acidic surface centers of the aerogel catalysts were investigated. Furthermore, the catalytic activity of this series of aerogels on the polycondensation of PBS polyester was measured so that we can compare and analyze the reaction kinetics of polycondensation of PBS catalyzed by TS91 and TBT. The experimental results show that:

(1) For TiO_2_-AG aerogel, the introduction of a small amount of Si can greatly improve the specific surface area, which may be due to the fact that the introduction of Si slows down the growth rate of TiO_2_ primary gel particles during the formation of the gel, leading to the formation of more titania–silica composite primary gel particles with smaller size.

(2) The surface acidity of the titania–silica composite aerogels prepared in the experiment is mainly L acidic centers. The introduction of a small amount of Si can greatly increase the surface L acidic centers’ content of the aerogel, which is due to the improvement of the specific surface area of the material. With the continuous increase of Si content, the L acidic centers’ density and total acid content on the surface of the aerogel decreased. Among them, TS91 had the highest total L acidic centers’ content reaching 370.29 μmol/g, which provides a large number of active sites to coordinate with carbonyl oxygen atoms. At the same time, a large number of alkoxy groups remained on the surface of the titania–silica composite aerogel, providing reaction sites for ligand-exchange reactions.

(3) The titania–silica composite aerogels show catalytic activity for the polycondensation of PBS. The intrinsic viscosity of the PBS prepared by TS91 with the highest activity is up to 1.74 dL/g, which is due to the high L acidic centers’ content on its surface. In addition, the addition of TS91 before or after esterification has no significant effect on its catalytic activity, which proves its excellent hydrolysis resistance. The terminal carboxyl content of PBS catalyzed by TS91 is only 25.45 mol/t, which is significantly lower than that of PBS catalyzed by TBT (51.68 mol/t).

In summary, the prepared titania–silica composite aerogel can effectively catalyze the polycondensation process of PBS. Compared with the traditional titanate catalyst, although the catalytic activity of titania–silica is slightly lower, the latter has better performance in terms of resistance to hydrolysis and inhibition of side reactions so as to effectively reduce the content of terminal carboxyl groups in the product. Our results show that the design of catalysts can provide a new route for improving the stability and quality of polyester products.

## Figures and Tables

**Table 1:** Surface structure data of composite aerogels with different Ti/Si ratios.

Samples	S_BET_ (m^2^/g)	S_ext_ (m^2^/g)	V_tot_ (cm^3^/g)	Pore Size (nm)
TiO_2_-AG	332.86	275.01	0.39	7.56
TS91	524.59	350.29	0.85	8.08
TS82	536.81	495.99	0.86	8.12
TS73	524.04	484.16	0.84	7.51
TS64	441.37	411.81	0.66	7.24
TS55	402.87	377.93	0.46	7.02

**Table 2:** Surface element content of composite aerogels with different Ti/Si ratios.

Samples	Element Content (atom.%)			
	Ti	Si	C	O
TS91	13.70	2.66	44.99	38.65
TS82	12.55	4.10	43.67	39.68
TS73	12.45	6.40	31.30	49.85
TS64	11.30	10.20	25.42	53.08
TS55	11.09	12.40	23.31	53.19

**Table 3:** Surface acid content and types of composite aerogels with different Ti/Si ratios.

Samples	L Acid Content (μmol/g)	B Acid Content (μmol/g)	L Acid Density (μmol/m^2^)
TiO_2_-AG	221.29	6.04	0.665
TS91	370.29	20.05	0.705
TS82	301.33	16.67	0.564
TS73	299.51	8.74	0.571
TS64	208.83	9.85	0.473
TS55	164.51	13.27	0.407

**Table 4:** Molecular weight information of PBS synthesized by different catalysts.

Catalyst	Introduction Stage of Catalyst	[η] (dL/g)	Mn (g/mol)	L	a	b	Terminal Carboxyl Content (mol/t)
TiO_2_-AG	After esterification	1.14	4.03 × 10^4^	90.69	−0.03	2.08	32.05
TS91	After esterification	1.74	7.72 × 10^4^	90.73	−0.17	1.97	25.45
TS82	After esterification	1.64	7.05 × 10^4^	90.70	−0.31	2.01	24.32
TS73	After esterification	1.61	6.85 × 10^4^	90.21	−0.27	2.67	25.31
TS64	After esterification	1.35	5.22 × 10^4^	89.15	−0.16	3.43	23.32
TS55	After esterification	0.93	2.94 × 10^4^	88.02	−0.20	4.51	26.44
TBT	After esterification	1.89	8.74 × 10^4^	88.14	−0.29	4.61	51.68
No catalyst	-	0.63	1.62 × 10^4^	82.21	−0.18	9.81	82.75
TS91	Before esterification	1.78	7.99 × 10^4^	91.03	−0.21	1.93	24.43
TBT	Before esterification	1.26	4.70 × 10^4^	88.26	−0.32	4.79	62.37

**Table 5:** Linear regression equations of different catalytic systems.

Polycondensation Temperature	Linear Regression Equations (TS91)	Linear Regression Equations (TBT)
220 °C	Mt = 2923 + 481t; K = 241	Mt = 2923 + 716t; K = 358
225 °C	Mt = 2923 + 656t; K = 328	Mt = 2923 + 933t; K = 467
230 °C	Mt = 2923 + 779t; K = 390	Mt = 2923 + 1042t; K = 521
235 °C	Mt = 2923 + 946t; K = 473	Mt = 2923 + 1053t; K = 527
240 °C	Mt = 2923 + 972t; K = 486	Mt = 2923 + 1180t; K = 590

## Data Availability

Not applicable.
